# Circular RNAs in osteoarthritis: indispensable regulators and novel strategies in clinical implications

**DOI:** 10.1186/s13075-021-02420-2

**Published:** 2021-01-12

**Authors:** Wenchao Zhang, Lin Qi, Ruiqi Chen, Jieyu He, Zhongyue Liu, Wanchun Wang, Chao Tu, Zhihong Li

**Affiliations:** 1 Department of Orthopedics, The Second Xiangya Hospital, Central South University, No.139 Middle Renmin Road, Changsha, Hunan 410011 P.R. China; 2 Hunan Key Laboratory of Tumor Models and Individualized Medicine, The Second Xiangya Hospital, Central South University, No.139 Middle Renmin Road, Changsha, Hunan 410011 P.R. China; 3 Department of Geriatrics, The Second Xiangya Hospital, Central South University, No.139 Middle Renmin Road, Changsha, Hunan 410011 P.R. China

**Keywords:** Circular RNAs, Osteoarthritis, Microenvironment, Chondrocytes, Extracellular matrix

## Abstract

Over the past decades, circular RNAs (circRNAs) have emerged as a hot spot and sparked intensive interest. Initially considered as the transcriptional noises, further studies have indicated that circRNAs are crucial regulators in multiple cellular biological processes, and thus engage in the development and progression of many diseases including osteoarthritis (OA). OA is a prevalent disease that mainly affects those aging, obese and post-traumatic population, posing as a major source of socioeconomic burden. Recently, numerous circRNAs have been found aberrantly expressed in OA tissues compared with counterparts. More importantly, circRNAs have been demonstrated to interplay with components in OA microenvironments, such as chondrocytes, synoviocytes and macrophages, by regulation of their proliferation, apoptosis, autophagy, inflammation, or extracellular matrix reorganization. Herein, in this review, we extensively summarize the roles of circRNAs in OA microenvironment, progression, and putative treatment, as well as envision the future directions for circRNAs research in OA, with the aim to provide a novel insight into this field.

## Background

Being prevalent mainly in aging, obese and post-traumatic population [1, 2], OA is one of the major sources of chronic joint pain and disability. It is characterized by dysfunctions of cartilage that involve in the apoptosis of chondrocytes, degradation of extracellular matrix (ECM), inflammation, etc. [3–5] OA has been recognized as a principal origin of socioeconomic burden coming in two forms. One is the excessive requirement for health-care system, another is the increase in medical costs. As it was concluded, during 1996 to 2005 in the United States, annual expenditures charged to insurers for women with OA was $4833 higher than that of without OA while the costs for man with OA surpassed those of their non-OA counterparts by $4036 [6]. Currently, the therapeutic methods for OA include the physical activities [7], acetaminophen and nonsteroidal anti-inflammatory drugs (NSAIDs), intra-articular steroid injections, and surgery [8]. Besides, novel treatments for OA such as the mesenchymal stem cells (MSCs) are also on the way [9]. However, the outcomes of OA patients are not satisfactory due to its inevitable destruction to the joint [10], thereby early diagnosis and treatment are of great importance for favorable prognosis.

Non-coding RNAs (ncRNAs) have been ascertained as the central regulators for progression of multiple diseases [11, 12]. Recently, several reviews have addressed the critical roles of ncRNAs in the pathogenesis, development and therapeutic potential of OA [13–15]. CircRNA is a special subset of ncRNA, discriminated by a covalently closed-loop structure, while without neither 5′-3′ polyadenylated nor polarity tail [16]. They are derived from the known protein-coding genes, comprising with one or more exons. Notably, they are exceptionally stable due to the loop structure [17]. In 1976, H L Sanger et al. identified the first circRNA, namely the viroids [18]. For a long time, circRNAs were regarded as transcriptional noises produced during abnormal splicing. Currently, with the application of high-throughput RNA sequencing (RNA-seq) in biological study, thousands of circRNAs have been newly identified and even functionally annotated in multiple physiological and pathological processes of eukaryotes, such as the progression of carcinoma [19, 20], inflammation [21], aging [22], infection [23], etc.

Recently, the relationship between OA and circRNAs has been partially elucidated. Emerging studies suggested that circRNAs could participate in the development of OA through mechanisms such as interfering chondrocytes proliferation and apoptosis [24], regulating ECM degradation [25], and inflammation [26]. Herein, in this review, we have summarized the roles of circRNAs in OA microenvironment, development, and putative treatment, as well as discussed the future directions toward circRNAs in OA.

## Biogenesis and mechanism of circRNAs

Derived from canonical splice sites of pre-mRNA [27], circRNAs are primarily produced by “back splicing”, a process which dependent on the cis-regulatory elements and trans-acting factors [28]. Based on the current evidences, three regulatory models are proposed underlying the biogenesis of circRNAs: (1) Spliceosome mediated back-splicing and ligation, which largely relies on the presence of canonical splice sites within the exons [29]. Spliceosome at the 5′ donor site attacking the 3′ acceptor site brings the downstream and upstream fragments into close proximity to form a circulation. The ligation, occurring on the same exon or between two exons, is also catalyzed by spliceosome [30]. A more recent study uncovered that the spliceosomes could assemble across the exon and take part in either the canonical linear splicing or back-splicing [31]. (2) Cis-elements involved circRNA biogenesis. Several circRNAs are processed from internal exons and flanking introns, in which the regulatory elements locating at introns flanking circularized exons are necessitated [32]. RNA paring formed in the introns flanking exons is deemed as a crucial regulator, which generates RNA duplexes that promotes the back-splicing [27]. The primary circulation produced by RNA paring finally forms into the circRNAs with intron included or excluded by back-splicing and alternative splicing [28]. Besides, RNA paring can happen in either the repetitive or the non-repetitive complementary sequences [33]. (3) RNA-binding protein (RBP) regulated circRNA formation. RBP can bind to the introns flanking exons to interfere with the normal splicing. A dimerization of RBPs is then formed and brings the downstream splice- donor site into close proximity with an upstream splice- acceptor site, which finally induces the formation of circRNA [34, 35].

**Fig. 1:**
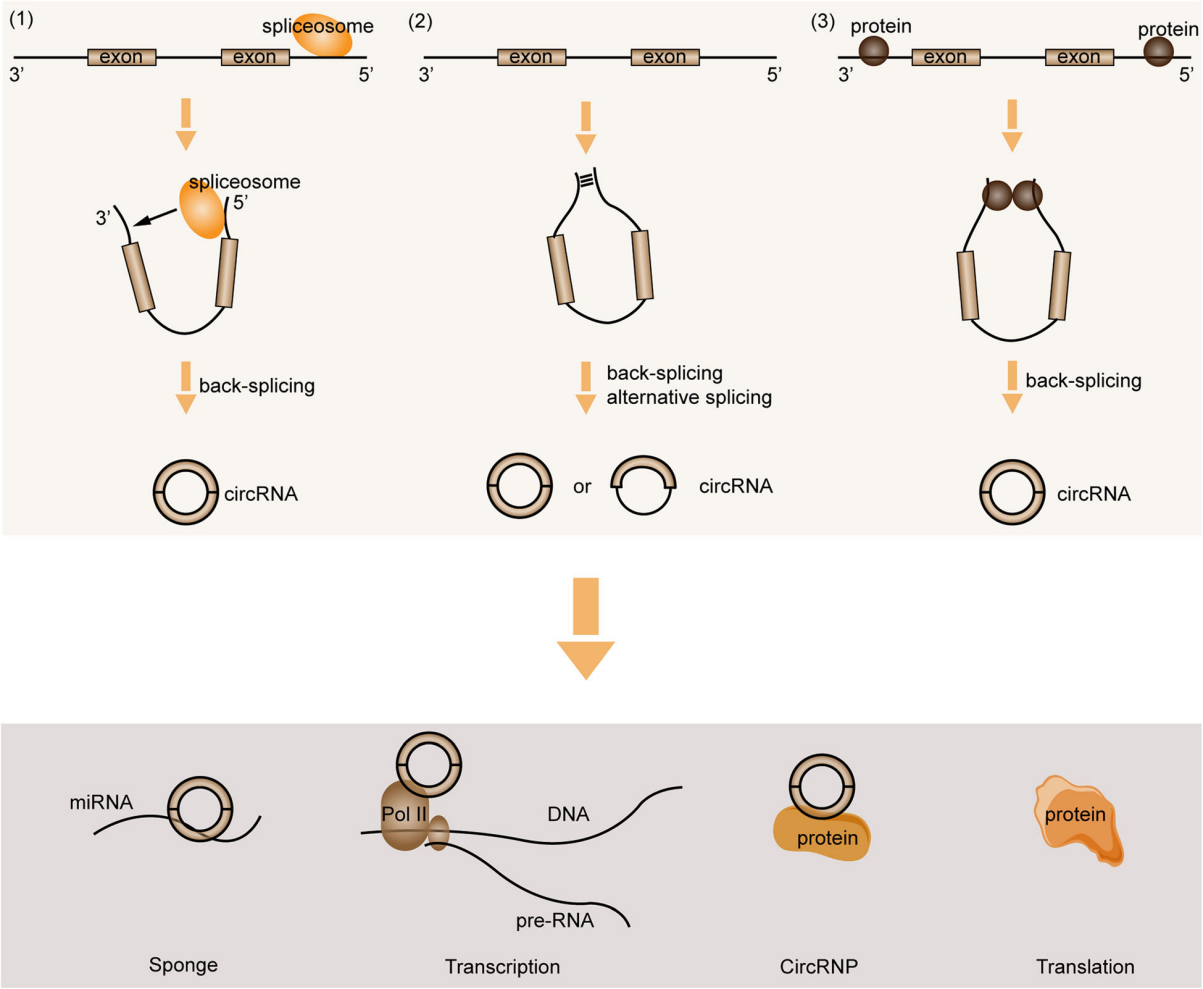
The biogenesis and general mechanisms of circRNAs. CircRNAs are generated through different ways: (1) Spliceosome mediated back-splicing and ligation; (2) Cis-elements involved circRNA biogenesis; (3) RNA-binding protein (RBP) regulated circRNA formation. Meanwhile, circRNAs may exert their functions through acting as miRNAs sponge, modulating the transcription, interplaying with protein directly, or translating into protein

As abovementioned, circRNAs are currently deemed as vital regulators in eukaryotes. Although the functions of overwhelming majority of circRNAs remain elusive, the present studies have showed that circRNAs regulate gene expression in multiple levels. Typically, circRNA can bind to miRNA as a sponge to perturb their expression, and thereby interfering the downstream mRNAs or proteins. For instance, ciRS-7 contains more than 60 miRNA-7 binding sites, and over 20,000 miRNA-7 molecules are bound by ciRS-7 per cell [36, 37]. Moreover, circRNAs engage in the regulation of gene transcription. Some circRNAs such as those derived from processed intron lariats (ciRNAs) [38] or from back-splicing containing the introns [39] are located in the nucleus. These circRNAs were reported to modulate the transcription of their parental genes in cis-regulatory way, which may be achieved by interacting with Pol II complex [39]. In addition, circRNAs can interplay with protein to produce specific circRNPs, which subsequently affecting functions of the associated proteins. It was proposed that there was a feedback loop between circRNAs and proteins from the same gene. Proteins could facilitate the biogenesis of circRNAs while circRNAs in turn control the expression of proteins [34]. Interestingly, circRNAs may also be translatable. However, due to the lack of 5′ end 7-methylguanosine (m7G) caps and 3′ poly(A) tails, circRNA translation is considered to happen in a novel way. It was suggested that some circRNA sequences could act as the internal ribosome entry sites (IRESs) to allow ribosomes binding to the translatable circRNAs [40]. For example, the translation of CircZNF609 in myogenesis [41], circMbL in fly [40], and circβ-catenin in liver cancer [42] are all happen in this manner. The detailed graphical description of these mechanisms is presented in Fig. 1.

## CircRNAs in microenvironment of OA

**Fig. 2:**
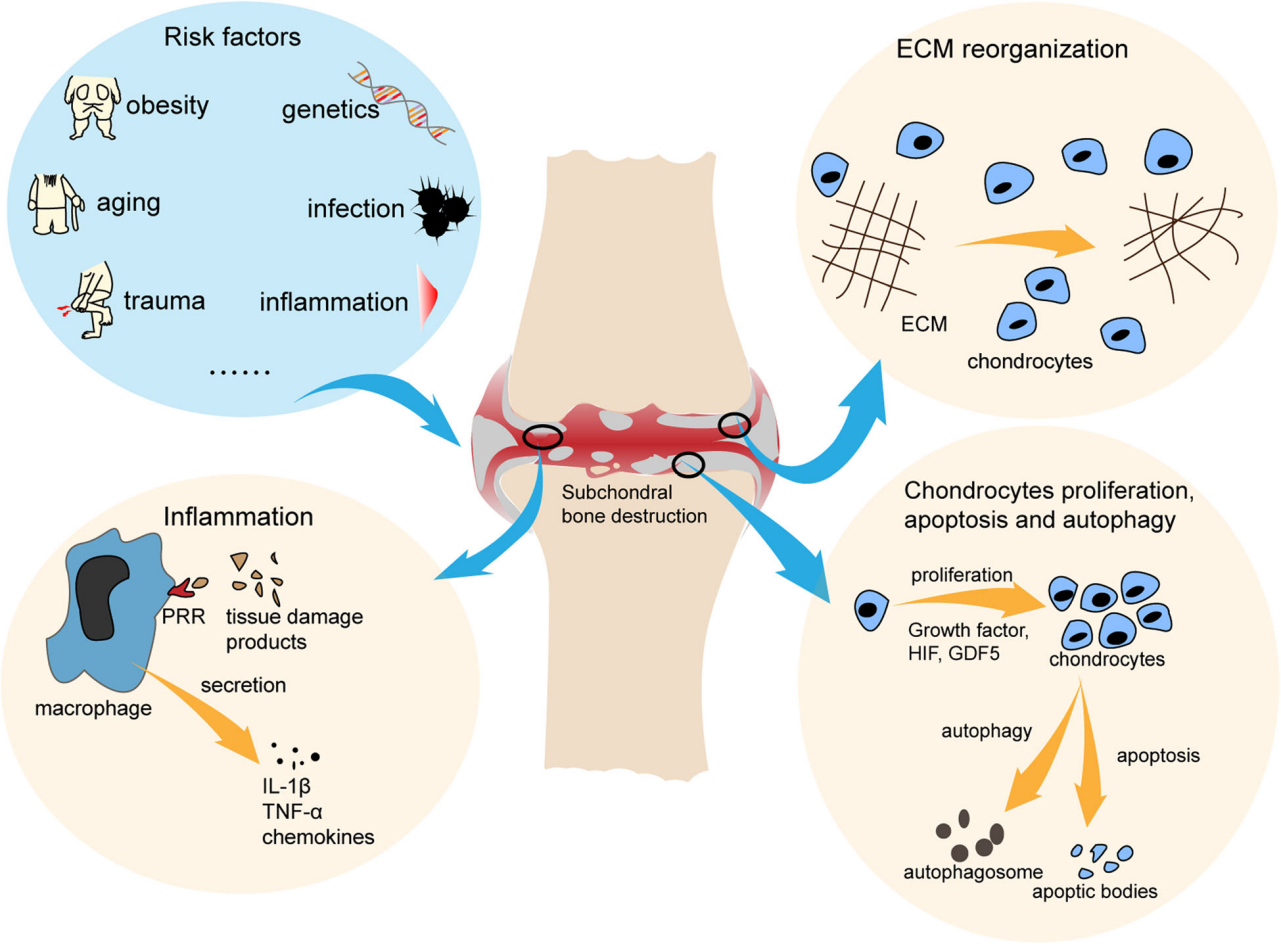
The OA microenvironment. Risk factors enter into the joint microenvironment and induce abnormities of cartilage. Firstly, the molecular composition and organization of ECM are changed to activate the proliferation and secretion of chondrocytes. The proliferating cells may produce high amounts of catabolic enzymes/ pro-inflammatory mediators which lead to chondrocyte apoptosis and ECM degradation. Innate immune cells such as the macrophages are motivated and the inflammatory responses then appear under the impact of various molecules. ECM: extracellular matrix, GDF5: growth differentiation factor 5, HIF: hypoxia-inducible factor

The pathogenesis and development of OA involve in multiple cells and molecules that construct the OA microenvironment, as shown in Fig. 2. Dysfunctions of articular chondrocytes, the main cellular component of cartilage, such as apoptosis [43] and autophagy [44] are considered to play an indispensable role during OA. During initial stage, the molecular composition and organization of ECM are altered while the cartilage surface remains intact [45]. Meanwhile, under the stimulation of those factors, the chondrocytes begin to acquire active proliferation ability and induce matrix secretion to repair the ECM. Molecules such as the growth factors [46], hypoxia-inducible factor 1α (HIF-1α) [47], and growth differentiation factor 5 (GDF5) [48] are found to participate in these processes. With the progression of OA, the articular microenvironment produces more factors to mitigate the deterioration. However, The proliferating cells may produce high amounts of catabolic enzymes/ pro-inflammatory mediators which finally lead to chondrocyte apoptosis and ECM degradation [49]. The cartilage is then disrupted and dysfunctional. Sequentially, the bone eburnation, osteophyte formation, muscle damage, and tendon destruction also appear [50]. Of note, inflammation also plays a role in the OA microenvironment, especially for the post-traumatic OA [51]. The products of tissue damage and stress recognized by the cell surface pattern-recognition receptors (PRRs) can trigger the inflammatory signal pathways such as the nuclear factor-κB (NF-κB) [52]. Multiple inflammation related molecules were found to be elevated in OA [53], including the interleukin 1β (IL-1β), tumor necrosis factor-α (TNF-α), chemokines, etc. Meanwhile, OA is usually companied by synovitis [54], even though its initiator remains unclear. Investigation of the OA synovium immune cell indicated that the CD14+ monocytes/macrophages, T cells, and mast cells were the dominating cell types [55], which implicated a role of innate immune system in OA inflammation [56].

As the indispensable component of OA microenvironment, ncRNAs have been demonstrated to participate in a multitude of OA related processes [57, 58]. Firstly, circRNAs constitute a large-scale subset of ncRNAs that exist in the OA microenvironment, which construct a regulatory network in OA progression. For instance, the proliferation, apoptosis, and degradation of articular chondrocytes are closely associated with abnormally expressed circRNAs [59, 60]. Zhu H et al. found that circGCN1L1 could promote the synoviocyte proliferation and chondrocyte apoptosis by sponging miR-330-3p [26]. However, the relationship between circRNAs and other cells in OA microenvironment such as the macrophage and fibroblast remain largely unknown.

Secondly, circRNAs are clarified as the vital regulators of cellular autophagy. Liang et al. have demonstrated that circCDYL could enhance the autophagic level in breast cancer cell by targeting miR-1275-ATG7, subsequently aggravating the tumor progression [61]. Meanwhile, circPAN3 was reported as a key mediator of drug resistance in acute myeloid leukemia by regulating autophagy and the AMP-activated protein kinase/ mammalian target of rapamycin (AMPK/mTOR) signaling pathway [62]. In addition, autophagy is also considered underlying the scenario of OA. Conclusive evidences were obtained from multiple studies that autophagy was increased in OA under the induction of catabolic and nutritional stresses, and that autophagy in turn regulated the expression of OA-like genes [63]. Moreover, the close interaction between autophagy and inflammation has been determined [64]. Notably, autophagy can perturb the inflammation of OA [65]. Therefore, circRNAs may act as critical mediators of autophagy in OA.

Besides, circRNAs also contribute to the innate immunity in OA [66]. Zhang and colleagues proposed that differential expression of 189 circRNAs were found between M1 and M2 macrophages [67]. Meanwhile, circ-RasGEF1B significantly affected the activation of lipopolysaccharide (LPS) exposed macrophages [68]. In addition, circ-ZC3H4 was proved as a crucial mediator of pulmonary macrophages activation induced by Silicon dioxide (SiO_2_) [69]. Further, the innate immunity related cells including monocytes/ macrophages and mast cell were disclosed to enrich in the OA microenvironment [55]. And it was noticed that innate immune response was in line with the development of OA [70]. Inhibition of macrophage activation could substantially reduce the inflammatory response and block the OA advancement in rat model [71]. Given these evidences, we envision that circRNAs are capable of interfering the progression of OA by affecting the innate immune system, which may pave a novel pathway to discover the underlying mechanism of OA.

## Multifaceted role of circRNAs in OA

### CircRNA profiles in OA

**Fig. 3:**
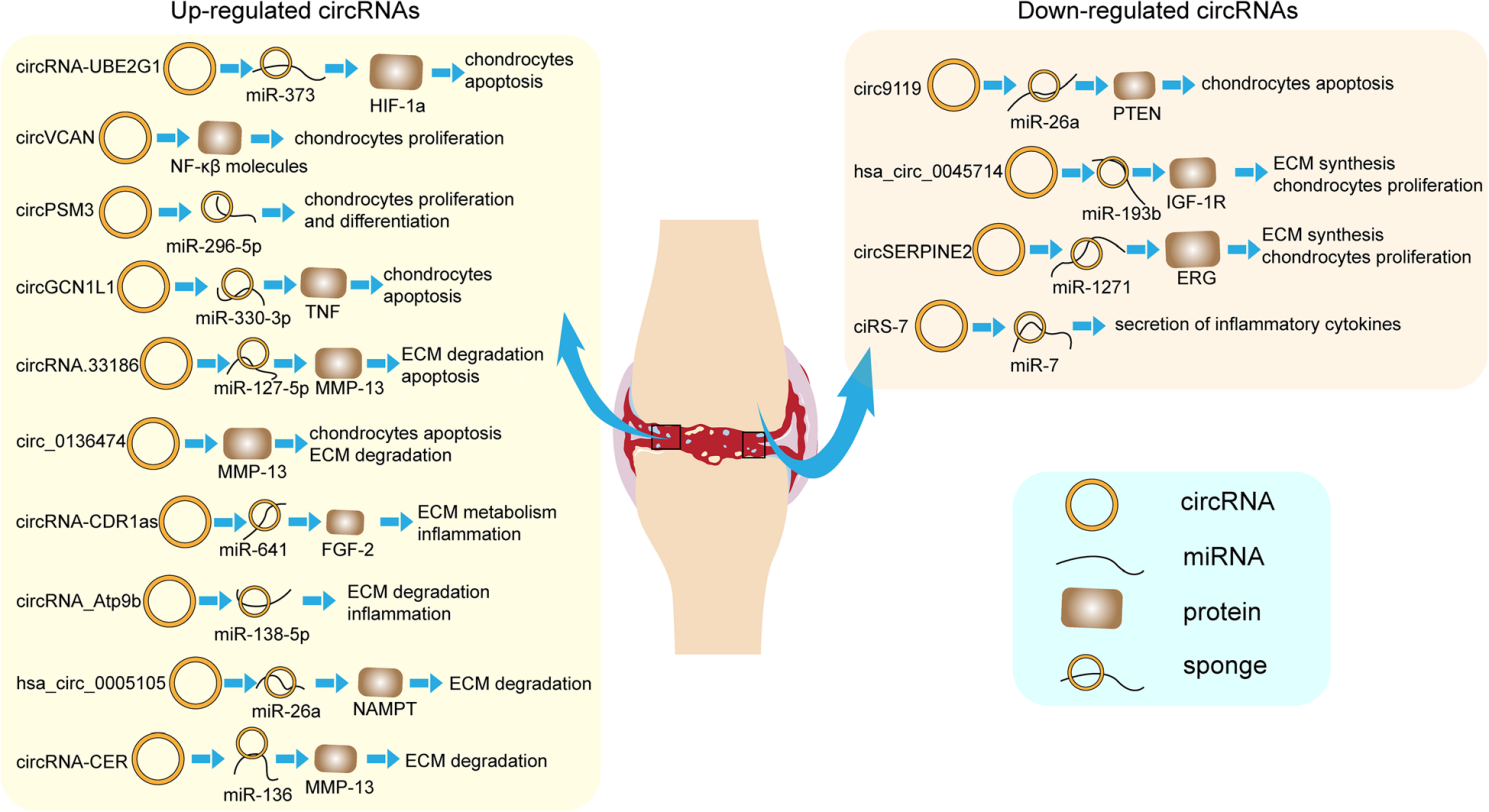
Detailed mechanism of circRNAs in OA. CircRNAs are either upregulated or downregulated in OA. The aberrant expressed circRNAs participate in the OA progression through binding to the miRNAs or proteins to interfere the downstream genes. Regulation of circRNAs on OA development involves in the chondrocytes proliferation and apoptosis, ECM metabolism, inflammation, etc. ECM: extracellular matrix, ERG: E26 transformation-specific (ETS)-related gene, HIF-1a: hypoxia-inducible factor-1a, IGF-1R: insulin-like growth factor 1 receptor, MMP-13: matrix metalloproteinase 13, NAMPT: nicotinamide phosphoribosyl transferase

Currently, it has been recognized that cartilage abnormality is significantly associated with the status of chondrocytes, including its differentiation [72], hypertrophy [73], apoptosis [43], regeneration [74], etc. In the past several years, there have been substantial studies toward these processes, in which the circRNAs took a big place. The aberrant changes of multiple circRNAs have been demonstrated in OA. A whole-transcriptome sequencing revealed that the expression of 42 circRNAs were altered in OA cartilage tissue compared with the normal cartilage [75]. Meanwhile, differential expressions of 1380 circRNAs were observed between OA chondrocytes and the counterparts [76]. Later, Xiao and colleagues unrevealed 197 differentially expressed circRNAs in OA knee condyle [77]. Furthermore, in OA mouse model induced by IL-1β, 119 upregulated and 136 downregulated circRNAs were also identified by the RNA-seq [78]. Additionally, expression level of 58 circRNAs were found to be altered in the temporomandibular joint (TMJ) OA tissue [79]. Collectively, the changing patterns of circRNAs indicate their potential functions in OA. Based on these genomic profiles, researches have been further performed toward these upregulated or downregulated circRNAs as follows. The detailed mechanisms are also displayed in Table 1 and Fig. 3.

**Table 1:** regulatory roles of circRNAs in OA

circRNAs	model/ system	Expression	Function	Pathway	Ref
circRNA-UBE2G1	Human cartilage/ C28/I2 cell line	↑	Reducing viability and promoting apoptosis of chondrocytes	circRNA-UBE2G1/ miR-373/ HIF-1a	[80]
circ_0136474	Human cartilage/ primary articular chondrocytes	↑	Inhibiting the proliferation and promoting the apoptosis of chondrocytes	circ_0136474/ MMP-13	[24]
circRNA-CER	Human cartilage/ primary articular chondrocytes	↑	Facilitating chondrocyte ECM degradation	circRNA-CER/ miR-136/ MMP-13	[81]
circVCAN	Human cartilage/ primary articular chondrocytes	↑	Promoting chondrocytes proliferation	circVCAN/ NF-κB	[60]
circPSM3	Human cartilage/ primary articular chondrocytes	↑	Enhancing chondrocytes proliferation and differentiation	circPSM3/ miRNA-296-5p	[59]
hsa_circ_0005105	Human primary articular chondrocytes (IL-1β stimulation)	↑	Promoting ECM degradation	hsa_circ_0005105/ miR-26a/NAMPT	[25]
circRNA-CDR1as	Human cartilage/ primary articular chondrocytes	↑	Modulating ECM metabolism and inflammation of chondrocytes	circRNA-CDR1as//miR-641/FGF-2	[82]
circRNA_Atp9b	Mouse articular chondrocytes (IL-1β stimulation)	↑	Promoting ECM degradation and inflammation of chondrocytes	circRNA_Atp9b/ miR-138-5p	[83]
circRNA.33186	Mouse DMM model/ primary chondrocytes	↑	Suppressing proliferation and promoting ECM degradation	circRNA.33186/ miR-127-5p/ MMP-13	[84]
circGCN1L1	Human TMJ synovial tissue/ primary synoviocyte/ primary chondrocytes	↑	Promoting chondrocytes apoptosis and synoviocyte proliferation	circGCN1L1/ miR-330-3p/ TNF-α	[26]
circ9119	Human cartilage/ SW1353 cell line	↓	Its overexpression protects chondrocytes from apoptosis	circ9119/miR-26a/PTEN	[85]
hsa_circ_0045714	Human cartilage/ primary articular chondrocytes	↓	Its overexpression promotes ECM synesis and chondrocytes proliferation	hsa_circ_0045714/ miR-193b/ IGF-1R	[86]
circSERPINE2	Human cartilage/ Rabbit ACLT model	↓	Its downregulation promotes the apoptosis and ECM degradation	circSERPINE2/ miR-1271/ ERG	[87]
ciRS-7	Human blood sample/ C28/I2 cell line	↓	Its downregulation promotes the secretion of inflammatory cytokines	ciRS-7/ miR-7	[88]

↑upregulation, ↓downregulation, *ECM* extracellular matrix, *ERG* E26 transformation-specific (ETS)-related gene, *HIF-1a* hypoxia-inducible factor-1a, *IGF-1R* insulin-like growth factor 1 receptor, *MMP-13* matrix metalloproteinase 13, *NAMPT* nicotinamide phosphoribosyl transferase

### Upregulated circRNA in OA

#### CircRNA-UBE2G1

A newly published study uncovered that the dysregulated circRNA-UBE2G1 accelerated OA progression [80]. By using Quantitative real-time reverse transcription PCR (qRT-PCR), the investigators found that circRNA-UBE2G1 level was considerably elevated in LPS-treated chondrocytes (C28/I2 cell line). Functional assays indicated that circRNA-UBE2G1 suppression could restore the viability of LPS-treated chondrocytes and decrease the level of pro-inflammatory cytokines. Mechanistically, circRNA-UBE2G1 acted as a competing endogenous RNAs (ceRNAs) and bound to miR-373, hereby increasing the expression of HIF-1α, a vital regulatory molecule in cell proliferation, survival [89], and synovial fibrosis under hypoxic conditions [90]. Thus, circRNA-UBE2G1 is an important regulator of OA advancement.

#### Circ_0136474

The expression of circ_0136474 in OA tissue was markedly increased compared with the normal counterpart according to the microarray analysis conducted by Li et al. [24]. Furthermore, cell counting kit-8 (CCK-8) assay, flow cytometry, and dual-luciferase reporter assay illustrated that overexpression of circ_0136474 in OA could inhibit the proliferation and promote the apoptosis of chondrocytes by competitive binding to miR-127-5p [24]. The immune-localization between circ_0136474 and matrix metalloproteinase 13 (MMP-13), a central regulator in cartilage degradation [89], has been confirmed by RNA fluorescence in situ hybridization (FISH) assy. Further exploration has ascertained that the suppression of miR-127-5p negatively regulated the MMP-13 expression. Taken together, enhancement of circ_0136474 in OA is a risk factor for cartilage damage and OA progress.

#### CircRNA-CER

CircRNA-CER, also known as circRNA 100,876, has been explored in OA and other human diseases. Previous studies demonstrated that aberrant circRNA-CER expression contributed to the proliferation and metastasis of gastric cancer [91], colorectal cancer [92], breast cancer [93], etc. Generally, circRNA-CER were reported to serve as ceRNA that bound to the corresponding miRNA.

In OA, the expression level of circRNA-CER was upregulated. Bioinformatics predicting the interaction between circRNA and miRNA suggested that circRNA-CER was closely correlated with miR-136 [81]. Their relationship was then validated by luciferase assay. Further, circRNA-CER inhibition reduced the expression of MMP-13 mRNA, and the co-transfection of miR-136 inhibitor and si-CER could reverse this effect [81]. Thus, it has been concluded that circRNA-CER could compete for miR-136 with MMP-13, consequently facilitating the process of chondrocyte ECM degradation.

#### CircVCAN

CircVCAN, locating at the chr5:83519349–83,522,309, has been partially studied in several human diseases. For instance, the expression level of CircVCAN in monocytes was considerably changed in sepsis, and its dysregulation was related to the condition of immunity [94].

Recently, circVCAN has been found to be highly expressed in OA violated cartilage tissue compared with that without OA destruction from meniscus injury patients [60]. Western blot and qRT-PCR showed that knockdown of circVCAN by siRNA could downregulate the mRNA and protein level of proliferating cell nuclear antigen (PCNA), and vice versa. Since PCNA is an important node in the cell proliferation regulatory network [95], the crosstalk between circVCAN and PCNA may suggest a role of circVCAN in proliferation as well. Besides, the flow cytometry connoted that chondrocytes tended to maintain at G0/G1 phase in the cell cycle and induce apoptosis when treated with si-CircVCAN. Moreover, NF-κB pathway was notably downregulated during circVCAN silencing. Additionally, when transfected with the NF-κB inhibitor, the effect of LV-CircVCAN (increasing circVCAN level) on chondrocytes proliferation and apoptosis was reversed. Therefore, CircVCAN could participate in the chondrocyte proliferation and apoptosis, partially via regulating the NF-κB pathway in OA.

#### CircPSM3

As validated by qRT-PCR, circPSM3 was upregulated in OA chondrocytes [59]. By contrast, miR-296-5p was found downregulated in OA, and luciferase reporter assay indicated that miR-296-5p was the target of circPSM3. MiR-296-5p could effectively enhance the cell proliferation [59]. Meanwhile, miR-296-5p inhibitor restored the cell proliferation and differentiation that was suppressed by si-circPSM3. Therefore, circPSM3 expedited chondrocytes proliferation and differentiation in OA via binding to miRNA-296-5p, whereby circPSM3 may serve as a potential therapeutic target for OA.

#### Hsa_circ_0005105

Hsa_circ_0005105 is a novel circRNA that located at chr5:134022479–134,023,989. Wu et al. reported that the expression level of hsa_circ_0005105 was markedly increased in IL-1β treated chondrocytes [25]. Western blot detection suggested that overexpression of hsa_circ_0005105 suppressed the expression of type II collagen (Col II) and aggrecan (AGG), the major components of hyaline (or intraarticular) cartilage [25]. Furthermore, miR-26a, the target gene of hsa_circ_0005105, was confirmed by dual-luciferase reporter assay. MiR-26a was found to inhibit the expression of nicotinamide phosphoribosyl transferase (NAMPT), an indispensable catabolic regulator in inflammatory pathway [95, 96]. However, the inhibition could be reversed by hsa_circ_0005105 upregulation [96, 97]. Hence, hsa_circ_0005105 modulated the progression of OA via a hsa_circ_0005105-miR-26a-NAMPT axis.

#### CircRNA-CDR1as

Locating at chrX:139865339–139,866,824, circRNA-CDR1as has a genomic length of 1485 nt. Based on the current studies, dysregulation of circRNA-CDR1 has been identified in a variety of physio-pathologic processes, such as the growth and chemo-sensitivity of carcinomas [98, 99], myogenic and osteoblastic differentiation [100, 101], and particularly, the progression of OA [82].

QRT-PCR examination illustrated that circRNA-CDR1as expression level in chondrocytes derived from OA cartilage was remarkably higher than that of the normal chondrocytes, and the deactivation of circRNA-CDR1as by siRNA boosted the expression of Col II, but reduced MMP-13 [82]. Additionally, down-regulation of circRNA-CDR1as minimized the level of inflammatory factor IL-6. Pull down assay further confirmed that circRNA-CDR1as could bind to miR-641. Fibroblast growth factor 2 (FGF-2) was then determined as the target of miR-641 by TargetScan website and dual luciferase reporter assay [82]. Further investigation discovered that by activating the mitogen-activated protein kinase kinase/extracellular regulated kinase (MEK/ERK) signaling pathway, circRNA-CDR1as/miR-641/FGF-2 axis facilitated the ECM degradation and inflammation of chondrocytes [82].

#### CircRNA_Atp9b

A study by Zhou et al. inferred a higher expression of circRNA_Atp9b in mouse OA chondrocytes [83]. Western blotting and qRT-PCR elucidated that knockdown of circRNA_Atp9b dramatically forced the synthesis of Col II, but diminished MMP-13 expression, inflammatory factors, IL-6 and cyclooxygenase-2 (COX-2) in chondrocytes [83]. Bioinformatics analysis and luciferase activity assay disclosed that circRNA_Atp9b functioned as a miR-138-5p sponge [83]. Meanwhile, transfection of miR-138-5p inhibitor could reverse the protective effect of circRNA_Atp9b suppression. Summarily, these findings manifested that circRNA_Atp9b was capable of modulating IL-1β-induced ECM synthesis by sponging miR-138-5p in OA chondrocytes [83]. Besides, circRNA_Atp9b was also proposed to regulate the inflammatory response in LPS induced MRC-5 cells by blocking the NF-κB and c-Jun N-terminal kinase (JNK) pathway [102].

#### CircRNA.33186

CircRNA.33186, derived from the Umad1 gene on chr6:8373906–8,427,185, was found to be expressed 4.83-fold higher in OA chondrocytes than the normal control [84]. In vivo study of OA mice models also disclosed the up-regulation of circRNA.33186 [84]. Moreover, the expression level of MMP-13 was significantly reduced while the Col II was up-expressed when the chondrocyte was transfected with si-circRNA.33186 [84]. CCK8 and 5-ethynyl-2′-deoxyuridine (EdU) assay illuminated that circRNA.33186 ablation could restore the proliferation of chondrocytes that was deactivated by IL-1β [84]. It was then figured out that miR-127-5p had a binding site for circRNA6.3318 and their relationship was further validated by luciferase screening assay [84]. Furthermore, miR-127-5p was clarified to target MMP-13. In particular, after co-transfecting with miR-127-5p inhibitor, the effects caused by silencing of circRNA6.3318 were significantly resumed [84]. In conclusion, the pathogenesis promotion effect of circRNA.33186 relied heavily on binding to miR-127-5p and acting as a sponge.

#### CircGCN1L1

Zhu et al. have investigated the role of a circGCN1L1 splicing variant that had an upregulated fold change of 153.3, the hsa_circ_0000448, in the progression of TMJ OA [26]. Mechanistic studies uncovered that circGCN1L1 in TMJ OA synovial cells and tissues could promote the chondrocytes apoptosis and synoviocytes proliferation. MiR-330-3p was found as the only miRNA that was predicted to have a binding site for circGCN1L1 in all of the three online databases (circular RNA interactome, StarBase, and RegRNA2.0) [26]. In vitro study displayed that expression level of miR-330-3p was negatively correlated with the circGCN1L1 expression. Further exploration ascertained that circGCN1L1 could target miR-330-3p as a sponge [26]. Moreover, miR-330-3p directly bound to TNF-α and restrained the secretion of matrix-degrading enzymes, including MMP3, MMP13 and a disintegrin and metalloproteinase with thrombospondin motifs-4 (ADAMTS4) [26]. From these data, it can be summarized that circGCN1L1-miR-330-3p-TNF axis exerted crucial function in TMJ OA modulation.

### Downregulated circRNA in OA

#### Circ9119

As shown by qRT-PCR, the expression level of circ9119 was significantly downregulated in OA tissue and IL-1β induced chondrocytes compared with the normal counterpart [85]. 3-(4,5-Dimethylthiazol-2-yl)-2,5-diphenyltetrazolium bromide (MTT) assay demonstrated that the suppression of chondrocytes proliferative activity was reversed after circ9119 overexpression treatment. Using bioinformatics analysis, miR-26a was found to be a target for circ9119, and the phosphatase and tensin homolog (PTEN) was targeted by miR-26a [85], which was further verified by luciferase reporter assay. The expression level of miR-26a was increased and PTEN was reduced in IL-1β induced chondrocytes, while their expression was restored after circ9119 overexpression [85]. Thus, these findings suggested that circ9119 accelerated the proliferation of IL-1β induced chondrocytes via binding to miR-26a, subsequently increasing the PTEN [103].

#### Hsa_circ_0045714

Hsa_circ_0045714, locating at chr17:73808192–73,809,959, was considerably down-regulated in OA tissue and TNF-a treated chondrocytes [86]. As revealed by luciferase reporter assay, miR-193b was targeted by hsa_circ_0045714, and their expression level was negatively correlated. Further investigations demonstrated that miR-193b could target insulin-like growth factor 1 receptor (IGF1R) and suppress its expression, while overexpression of hsa_circ_0045714 antagonized this repressive effect [86]. Moreover, IGF-1R could increase the secretion of Col II and AGG in chondrocytes while miR-193b restrained their expression. Similarly, the inhibition effect of miR-193b could be reversed by hsa_circ_0045714 overexpression [86]. Besides, IGF-1R and hsa_circ_0045714 could accelerate the proliferation of chondrocytes, while miR-193b enhanced cell apoptosis [86]. Collectively, these results indicated that hsa_circ_0045714 regulated the ECM synthesis and proliferation of chondrocytes by abolishing miR-193b expression, whereby promoting the IGF-1R gene.

#### CircSERPINE2

CircSERPINE2 is located at chr2:224856519–224,866,639 in human genome. Detection in 10 human OA cartilages and 10 controls from different donors illustrated that circSERPINE2 expression was down-expressed in OA sample [87]. Knockdown of circSERPINE2 was found to accelerate the apoptosis and motivate the expression of MMP3, MMP13 and ADAMTS4 in human chondrocytes and SW1353 cells (chondrosarcoma cell lines). Luciferase assay has verified that miR-1271 was a target for circSERPINE2. Meanwhile, chondrocytes apoptosis was significantly restored when the si-circSERPINE2 was co-transfected with miR-1271 inhibitor. Moreover, the E26 transformation-specific (ETS)-related gene (ERG) was validated as the target gene of miR-1271 [87]. The in vivo study using ACLT rabbit model further implied that circSERPINE2 could alleviate the progression of OA [87]. In summary, overexpression of circSERPINE2 protected chondrocytes from apoptosis and ECM degradation via miR-1271-ERG pathway.

#### CiRS-7

CiRS-7, also termed as CDR1as, is originated from the antisense orientation transcription of CDR1 gene [104]. It is a circular miR-7 inhibitor with more than 70 binding sites [104], and the ciRS-7/miR-7 axis has been identified to play a regulatory role in many diseases. It was reported that the ciRS-7/miR-7/NF-kβ axis could significantly interfere the invasion, proliferation, and migration of non-small cell lung cancer cells [105]. Further, it could impact the insulin secretion by targeting Myrip and Pax6 [106].

In the plasma of OA patients, ciRS-7 expression was downregulated while miR-7 expression was upregulated compared with the healthy subjects [88]. Further investigation has revealed that knockdown of ciRS-7 could promote the secretion of inflammatory cytokines such as IL-6 and IL-8 [88]. Flow cytometry unraveled that ciRS-7 inhibition and miR-7 activation induced the pro-apoptotic effects in OA cells. In conclusion, these findings indicated that ciRS-7/miR-7 axis exerted important function in the regulation of OA.

## Potential clinical utilizations of circRNAs in OA

Considering the pivotal roles of circRNAs in the progression and development of OA, it has been speculated that circRNAs are of great importance in clinical application like other ncRNAs [5, 107]. Particularly, there has been increasingly more attention on changes of circRNAs in body fluid such as the peripheral blood and synovial fluid since they are easy to acquire and detect. Wang et al. have uncovered that hsa_circ_0032131 level in peripheral blood of OA patients was differentially expressed and could serve as a biomarker for diagnosis [108]. The value of area under curve (AUC) for hsa_circ_0032131 was 0.8455. In parallel to this, peripheral blood hsa_circ_0020014 was also illuminated to be a potential biomarker for OA diagnosis with an AUC of 0.6415 [109]. Similarly, in blood sample of OA patients, ciRS-7 expression level was notably decreased, which indicated the potential of ciRS-7 to be a diagnostic biomarker [88]. Likewise, circRNA expression profiles in synovial fluid of OA patients have also been carried out by microarray analysis [110]. Results showed that the expression level of three circRNAs (hsa_circ_0104595, hsa_circ_0104873, and hsa_circ_0101251) could distinctly distinguish OA patients from their normal counterpart, with the AUC value of 0.708, 0.683, and 0.754, respectively. Therefore, circRNAs may act as promising biomarkers for the prediction of OA (Table 2).

**Table 2:** Potential clinical applications of circRNAs in OA

CircRNA	Sample	Detection method	Expression	Parameter	Application	Ref
hsa_circ_0032131	peripheral blood	qRT-PCR	↑	AUC (0.8455)	diagnostic biomarker	[108]
hsa_circ_0020014	peripheral blood	qRT-PCR	↓	AUC (0.6415)	diagnostic biomarker	[109]
ciRS-7	peripheral blood	qRT-PCR	↓	NA	diagnostic biomarker	[88]
hsa_circ_0104595	synovial fluid	microarray analysis, qRT-PCR	↑	AUC (0.708)	diagnostic biomarker	[110]
hsa_circ_0104873	synovial fluid	microarray analysis, qRT-PCR	↑	AUC (0.683)	diagnostic biomarker	[110]
hsa_circ_0101251	synovial fluid	microarray analysis, qRT-PCR	↑	AUC (0.754)	diagnostic biomarker	[110]

↑upregulation, ↓downregulation, *AUC* area under curve

Moreover, circRNAs can also act as the therapeutic targets for drugs as outlined above, yet there is currently no such drug in OA. Some manipulation strategies have been developed to inhibit or enhance the expression level of circRNAs. The initial method to suppress circRNAs expression is siRNA which complements to the back-splice site to interfere with the circRNAs production [111]. Genome-editing tools such as CRISPR/Cas9 also have been used in removal of functional circRNAs [112], although their application in human body is still in fierce controversy.

Further, several newly established strategies for diseases treatment may expand the therapeutic application of circRNAs in OA. For instance, circRNAs are found in extracellular vesicles (EVs) that can modulate the progress of diseases by transporting their cargo (ncRNA, protein, mRNA, lipid, etc.) [5, 113]. Since EVs are also the important regulators in cartilage homeostasis and osteoarthritis [114], circRNAs may partially exert their function via the EVs transportation system. In fact, some exosomal ncRNAs such as miRNAs have been illustrated to take part in the regulation of OA. As an example, exosomal miR-95-5p can protect cartilage from harassment of OA through directly targeting histone deacetylase 2/8 (HDAC2/8) [115]. Moreover, the emerging role of other exosomal ncRNAs including the lncRNAs has been illustrated in musculoskeletal disorders [5, 116]. In addition, EVs have shown great potential as the vehicle to selectively deliver drugs into specific site of tissue [117]. By delivering the engineered RVG-circSCMH1-EVs to the brain of stroke mice and monkeys, Li et al. have validated that exogenous circRNAs delivery by EVs could improve the outcome of stroke [118]. Accordingly, delivering effective exogenous circRNAs into OA site by EVs is a novel direction for OA treatment.

Moreover, mesenchymal stem cells (MSCs) are another potential treatment option for OA that have sparked intensive research interest in recent years [119]. Derived from human organs such as the bone marrow and adipose, MSCs are multipotent progenitor cells that are capable of differentiating into different lineages including chondrocytes to repair tissue damage [120, 121]. Combining with scaffolds, MSCs can promote the regeneration of articular cartilage [122]. Additionally, the MSCs derived exosome can promote the proliferation and suppress the apoptosis of chondrocytes [123], as well as alleviate the inflammation response in OA [124]. Of note, the expression level of a large diversity of circRNAs have been changed during chondrogenic differentiation of MSCs [125]. Among them, CircRNA-CDR1as has been validated to maintain the differentiation and proliferation of human umbilical cord MSCs [126]. Thus, circRNAs may be utilized as the regulator to determine the differentiation trend and speed during the MSCs therapy, which pave the way for customized treatment.

## Methods for circRNA research

Over the past decades, emerging technologies have been developed and adopted to explore the presences and functional implications of circRNAs even though the current methods are still of limitations. Here we have summarized and discussed the existing approaches as well as their deficiencies in circRNA research.

### Genome-wide circRNA profiling

Conventional genome-wide sequencing is not suitable for circRNAs because of their specific circular structure. To figure out this limitation, several circRNA collection and purification methods such as circRNA enrichment using RNase R have been invented [127] to remove linear RNAs ahead of sequencing. Moreover, the identification of specific circRNAs relies on recognition of their back-spliced junction (BSJ) reads, so multiple annotation-dependent approaches (such as MapSplice [128], CIRCexplorer [129], KNIFE [130], etc.) and algorithms (such as fnd-circ, segemehl [131], CIRI [132], etc.) for globally detection of circRNAs based on RNA-seq data also have been created. However, using different algorithms for circRNA prediction may lead to discrepant results, therefore it was suggested that several algorithms should be combined together to ensure the reliability [133].

### Experimental detection and validation of circRNAs

Several options are available for experimental validation of circRNAs, containing PCR, gel electrophoresis, Sanger sequencing, FISH, and Northern Blot. PCR is currently one of the most widely used method for fast detection of circRNAs. Firstly, primers that spanning the splice site are designed in a convergent direction. Then, the semiquantitative or quantitative PCR is conducted accordingly. However, PCR results of circRNAs are easy to be influenced by the linear RNAs that have the same sequences with the circRNAs reverse transcriptional fragments. Thus, subsequent gel electrophoresis and Sanger sequencing are recommended to further validate the PCR product [134]. Another experimental validation approach of circRNAs is Northern Blot, which represents as the golden standard [135]. CircRNAs detection can be achieved by either short probes spanning the BSJ or longer probes overlapping as much as an entire circularized exon [135]. This method is of high specificity and great versatility, even though it is laborious and time-consuming.

Meanwhile, the subcellular location of circRNAs is usually explored by FISH, a technique using probe to detect specific site in RNAs [136]. A fluorescently labeled junction specific probe is designed to discover and visualize circRNAs in cells. However, the expression level of a majority of circRNAs are relatively low in cells, which makes it difficult to be detected by fluorescence. Moreover, circRNAs fluorescent signal can be disturbed by the linear trans-spliced RNA isoforms. To overcome this limitation, pre-treating RNAs with RNase R has been applied [39].

### Expression interference of circRNAs

To explore functions of circRNAs, the loss and gain of function by manipulating its expression are necessary. Similar to the linear RNAs, suppression of circRNAs can be accomplished by RNAi technology. Nevertheless, the specific siRNA or shRNA must target the BSJ sites of circRNAs to avert silence of its cognate linear RNAs. This yields a limitation that we cannot design manifold RNAi fragments to overcome the off-target effect [127]. In addition, CRISPR/Cas9 is a potential method to achieve circRNA knockdown, which is fulfilled by large fragments deletion. Piwecka et al. have generated a circRNA CDR1as KO animal model by removing the entire CDR1as producing region [112]. However, CRISPR/Cas9 is still an unmatured tool for circRNAs suppression, and it inevitably impacts the expression of linear RNAs. Meanwhile, the entire genomic removal of gene locus may have enigmatic effects on cells.

Overexpression of circRNAs remains challenging. Complementary sequence-mediated RNA circularization is a classical manner for circRNA upregulation, which is implemented by a constructed plasmid compromising the circRNA-producing exons, flanking intronic sequences and intrinsic complementary sequences. With well-designed vector, the expression efficiency is high; however, the presence of non-specific linear RNA isoforms is common. Thomas et al. have designed a vector consisting of the upstream intron, downstream intron fragment (reverse complement with upstream intron), and the sequence for circRNA circularization that can be cloned between upstream and downstream sequences [36]. Compared with traditional cloning, this vector is of higher efficiency for circRNA amplification. Another way for circRNAs overexpression is to modulate the Quaking level or insert Quaking binding motifs into introns [35]. Since Quaking is a dimer that can bind to two separated regions of a single RNA molecule, it is possible that Quaking promotes the biogenesis of circRNAs. However, this technology has not been well developed currently.

### Synthesis of circRNAs in vitro

CircRNAs production in vitro is indispensable for the structural and functional studies. To acquire a circRNA, the linear RNAs generated from chemical synthesis or enzymatic transcription are ligated using various protocols [137] (such as chemical, enzymatic, or DNAzyme-supported protocols). The chemical synthesis is capable of producing RNAs with different backbone, linkages, and base modifications, which allows a wide range of customization. As another choice, the enzymatic protocols represent advantages in producing long RNAs that may exceed 1000 bp in length. However, it lacks of the possibility for site-specific modification [138].

In summary, technologies for circRNAs research have been partially developed. Multiple aspects of circRNAs (such as the presence validation, structure, loss and gain of function, and artificial synthesis) have been explored using these strategies, which has deepened our understand toward the role of circRNAs in diseases. Nevertheless, some methods remain unmatured in circRNAs study such as the CRISPR/Cas9. Advances of scientific study largely relies on the evolution of technology; therefore, novel insight of circRNAs may continuously uncovered with employment of new experimental assay.

## Conclusions and perspectives

CircRNAs have emerged as the novel and key modulators in OA with great potential serving as the diagnostic biomarkers or therapeutic regulators. The contributions of circRNAs in cartilage homeostasis, microenvironment, progression, and putative therapy for OA have been discussed in this review. Moreover, it has been highlighted that circRNAs may be combined with some novel therapeutic strategies such as the EVs drug delivery system and MSCs treatment to extend their therapeutic applications.

Nevertheless, the current studies toward circRNAs and OA are still of limitations. On the one hand, as outlined above, almost all of the researches focus on the “sponge” function of circRNAs in OA while other mechanisms such as the transcriptional regulation and interaction with RBP remain elusive. On the other hand, studies concerning the clinical applications of circRNAs in OA are limited to the diagnostic biomarker and possible therapeutic target, many novel directions are not explored yet.

Nevertheless, studies have uncovered novel interactions between circRNAs and multiple biological and pathological processes. For example, N6-methyladenosine (m^6^A) modification [139], the most abundant post-transcriptional RNA modification in mammals [140], has been demonstrated to regulate circRNA translation, degradation, export, etc. [141]. Chen and colleagues have found that m^6^A modification of circNSUN2 could enhance the liver metastasis of colorectal cancer by interfering the export of circNSUN2 [142]. Based on this, m^6^A modification of circRNAs may underlie the progression of OA. Interestingly, m^6^A modification of circRNAs also participates in the regulation of innate immunity that plays a crucial role in OA microenvironment. The innate immunity can be activated by endogenous and exogenous RNAs [143]. In contrast, m^6^A modification has also shown to suppress the innate immunity through YTH domain family member 2 (YTHDF2) that sequestered and blocked endogenous m6A-circRNA from activating the RIG-I antiviral pathway [144]. These findings indicate that m^6^A modified circRNAs may play a major part in OA progression.

Besides, the controlled generation of circRNAs is another hotspot, although there has been no study exploring it in OA. The artificial circulation can be formed in vivo or in vitro through different strategies such as the chemical ligation method, tornado method, and tRNA splicing machinery [145]. For instance, in vitro, the desired circRNAs were constructed by cloning the target sequence into an artificial exon that is flanked by repeated complementary intron, followed by transfection of plasmid into cells [146]. The artificial produced circRNAs may be useful tool in the targeted therapy of diseases. Jost and colleagues have designed a circRNA molecule containing the binding sites for miRNA-122, which was effective in inhibiting the secretion of hepatitis C viral proteins [147]. Since circRNAs are the key regulators of OA, this technology may provide new possibility for the treatment of OA.

Collectively, circRNAs are multifaceted regulators in OA progression, and it is beyond doubt that these circRNAs play important roles in exploring novel diagnostic and therapeutic strategies for OA. However, evidences about what properties make them improved over other biomarkers and the efficacy of them are scarce. Future studies are supposed to address these issues.

## Data Availability

The datasets used and/or analyzed during the current study are available from the corresponding author on reasonable request.

## Abbreviations

circRNAs: circular RNAs
OA: osteoarthritis
ECM: extracellular matrix
NSAIDs: nonsteroidal anti-inflammatory drugs
MSCs: mesenchymal stem cells
ncRNAs: Non-coding RNAs
RBP: RNA-binding protein
m7G: 7-methylguanosine
IRESs: internal ribosome entry sites
HIF-1α: hypoxia-inducible factor-1α
GDF5: growth differentiation factor 5
NF-κB: nuclear factor-Κb
IL-β: interleukin 1β
LPS: lipopolysaccharide
AMPK: autophagy and the AMP-activated protein kinase
mTOR: mammalian target of rapamycin
MEK: mitogen-activated protein kinase kinase
ERK: extracellular regulated kinase
JNK: c-Jun N-terminal kinase
COX-2: cyclooxygenase-2
TNF-α: tumor necrosis factor-α
CCK8: cell counting kit-8
EdU: 5-ethynyl-2′-deoxyuridine
TMJ: temporomandibular joint
ceRNAs: competing endogenous RNAs
MMP-13: matrix metalloproteinase 13
RNA-FISH: RNA- fluorescence in situ hybridization
Col II: type II collagen
NAMPT: nicotinamide phosphoribosyl transferase
FGF-2: fibroblast growth factor 2
MTT: 3-(4,5-Dimethylthiazol-2-yl)-2,5-diphenyltetrazolium bromide
PTEN: phosphatase and tensin homolog
ADAMTS4: a disintegrin and metalloproteinase with thrombospondin motifs-4
IGF1R: insulin-like growth factor 1 receptor
AUC: area under curve
EVs: extracellular vesicles
HDAC: histone deacetylase
MSCs: mesenchymal stem cells
m^6^A: N6-methyladenosine
YTHDF2: YTH domain family member 2
